# Supplementary material for: Intervention Programme Based on Self-Determination Theory to Promote Extracurricular Physical Activity through Physical Education in Primary School: A Study Protocol
Source: Children (Basel). 2023 Mar 3;10(3):504. doi: 10.3390/children10030504 (PMC10047147; doi:10.3390/children10030504)
Supplement: Supplementary file 1 [file children-10-00504-s001.zip › children-2213251-supplementary.pdf]

Supplementary material

Table S1. Check List of Need Support Behaviours

The Physical Education teacher…	**YES**	**NO**	**Remarks**
1. Encourages the autonomous practice of physical and sporting activities in their free time.			
2. Encourages and gives positive feedback when pupils do physical activities and sports outside school.			
3. Encourages pupils to do physical and sporting activities with family and friends in their free time.			
4. Offers novel options for after-school physical and sporting activities.			
5. Cares about, listens to and gives advice to pupils about physical and sporting activities undertaken independently in their free time.			
6. Shows confidence in the pupils' ability to do physical and sporting activities in their free time.			
7. Encourages sharing with the class the experiences of physical and sporting activities carried out in company.			
8. Introduces new physical and sporting activities to be undertaken in pupils' free time.			
General remarks:			
